# Evolution of Flavanol Glycosides during Red Grape Fermentation

**DOI:** 10.3390/molecules23123300

**Published:** 2018-12-12

**Authors:** Marie Zerbib, Guillaume Cazals, Marie-Agnès Ducasse, Christine Enjalbal, Cédric Saucier

**Affiliations:** 1 SPO, Univ Montpellier, INRA, Montpellier Supagro, 34000 Montpellier, France; marie.zerbib@umontpellier.fr; 2 IBMM, Univ Montpellier, CNRS, ENSCM, 34000 Montpellier, France; guillaume.cazals@umontpellier.fr (G.C.); christine.enjalbal@umontpellier.fr (C.E.); 3 UMT Minicave, Institut Français de la Vigne et du Vin, 11430 Gruissan, France; Marie-Agnes.ducasse@vignevin.com

**Keywords:** red grape fermentation, flavan-3-ol, glycoside, diglycoside, multiple reaction monitoring (MRM), mass spectrometry

## Abstract

Monomeric and dimeric flavanol glycosides were quantified by UHPLC-MRM in Syrah (SYR) and Grenache (GRE) grapes and in their corresponding wines for the first time. Quantities were extremely variable depending on grape tissue (seeds or skins) and during fermentation. Overall, 22 monomeric and dimeric mono- and diglycosides were determined with concentrations ranging from 0.7 nanograms to 0.700 micrograms per gram of grape tissue, and 0 to 60 micrograms per liter for wines. The evolution of the glycosides’ composition during winemaking suggests that almost all these compounds originate in the grapes themselves and display different extraction kinetics during winemaking. One isomer of the monomeric (epi) flavanol monoglycosides seemed to be biosynthesized by yeasts during wine fermentation. The sharp decrease in concentration of some isomers at the late stages of fermentation or after pressing suggests that some grape glycosidase activities convert these compounds into non-glycosylated flavanols.

## 1. Introduction

Polyphenols are secondary metabolites present in a wide variety of natural products such as grapes, tea, and cocoa [1]. They have health properties such as antioxidant and anti-inflammatory activities and play a role in protecting against cardiovascular diseases [2,3]. Polyphenols are also involved in plant defense mechanisms [4,5], and have a major impact on the quality of wine organoleptic properties such as color [6,7], astringency, and bitterness [8,9].

Flavan-3-ols belong to the flavonoid class of phenolic compounds. Some studies have been carried out on how their composition impacts the organoleptic properties of different grape tissues and wines [10,11]. Flavanols found in grape skin consist of (epi) catechin, (epi) catechin gallate, and epigallocatechin monomers and their corresponding polymers (procyanidins and prodelphinidins) [12]. In seeds, the flavanol content includes (epi) catechin, epicatechin gallate, and procyanidins [13,14]. 

**Figure 1:**
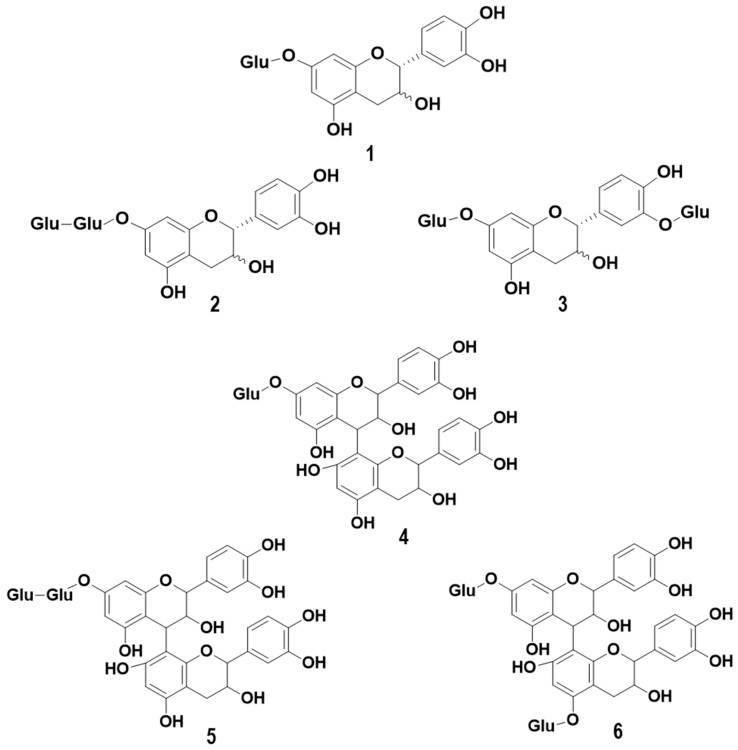
Examples of monomeric (epi) catechin mono glucoside (**1**), monomeric (epi) catechin diglycosides (**2** and **3**), dimeric (epi) catechin monoglycoside (**4**), and dimeric (epi) catechin diglycosides (**5** and **6**).

Monomeric and dimeric glycoside forms of monomeric and dimeric flavanols have been identified in different varieties of grapes and wine [15,16]. Some examples of their structures are presented in Figure 1. Studies have been reported on their in vivo biological properties in plants or animals. For example, experiments on rats showed that catechin glucosides increase the absorption of the flavanols mentioned above [17]. Furthermore, it has been reported that glucosylated catechin, as opposed to its aglycone equivalent, confers greater resistance to light radiation and tyrosinase activity [18]. Dixon and collaborators found that in plants, the over-expression of glucosyltransferase UGT72L1 in the seed coat of *Medicago truncatula* increased the accumulation of proanthocyanidins (PA) and epicatechin 3′-*O*-glucoside, suggesting that this flavanol glycoside may be involved in the biosynthesis of PA [19]. 

Changes in flavanol concentration during fermentation depend on the percentage of alcohol and the duration of contact between the skins and wine (maceration). Their concentration increases to reach a maximum after about six days of fermentation and then decreases at the end of alcoholic fermentation (AF). More flavan-3-ols are extracted from grape skins than from seeds during AF [11]. The principal objective of this work was to study, for the first time, the extraction kinetics of mono- and dimeric flavanols and mono- and diglycosides in grapes and during winemaking. 

## 2. Results and Discussion

**Figure 2:**
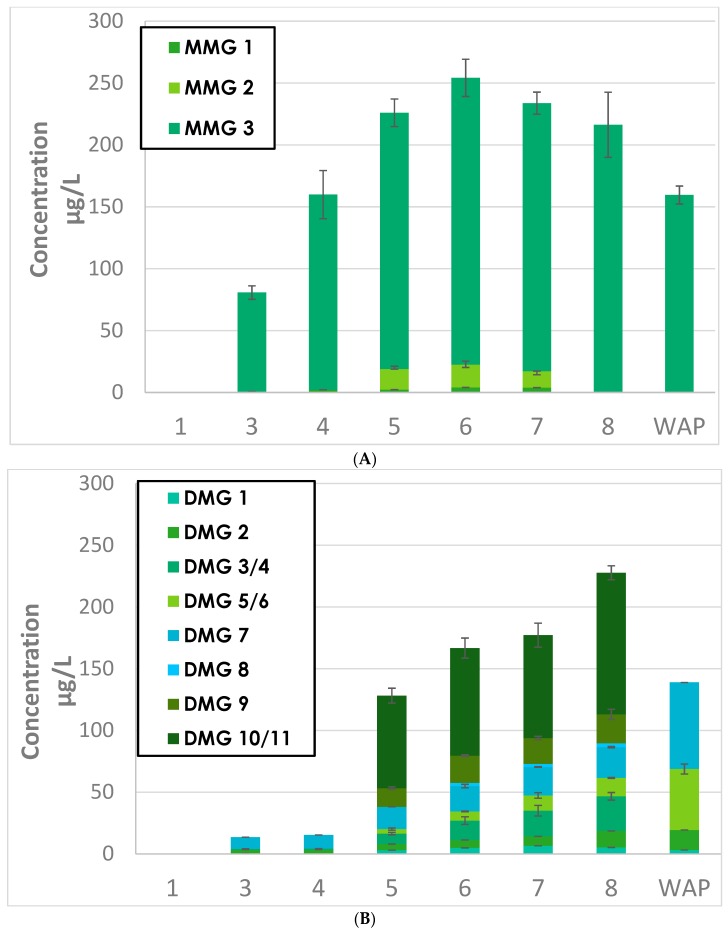
Evolution of (**A**) monomers of (epi) catechin monoglycosides (MMG) and (**B**) dimers of (epi) catechin monoglycosides (DMG) isomers during Grenache (GRE) fermentation. WAP = wine after pressing.

**Figure 3:**
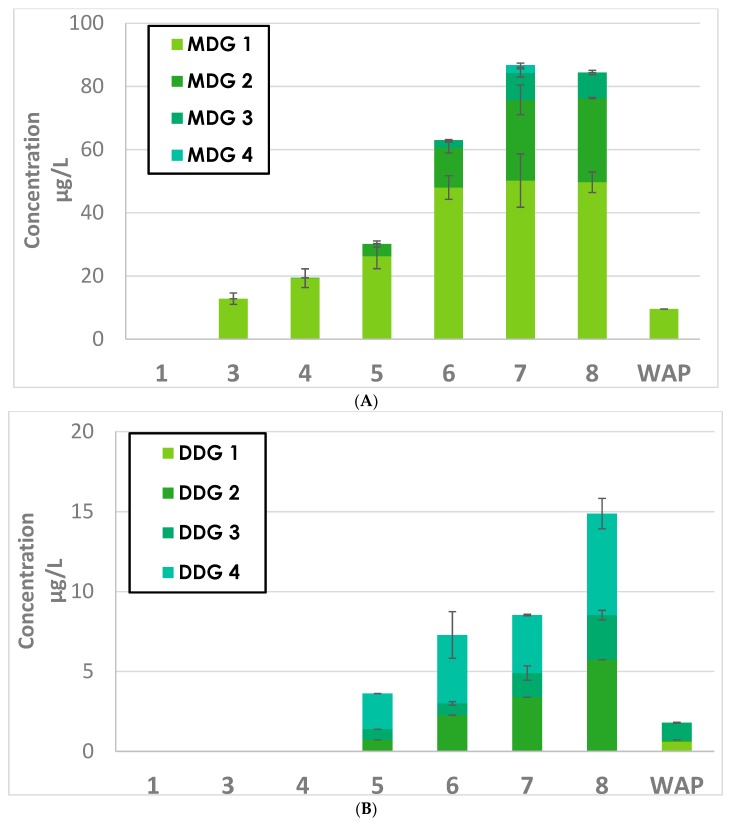
Evolution of (**A**) monomers of (epi) catechin diglycosides (MDG) and (**B**) dimers of (epi) catechin diglycosides (DDG) during GRE fermentation.

**Figure 4:**
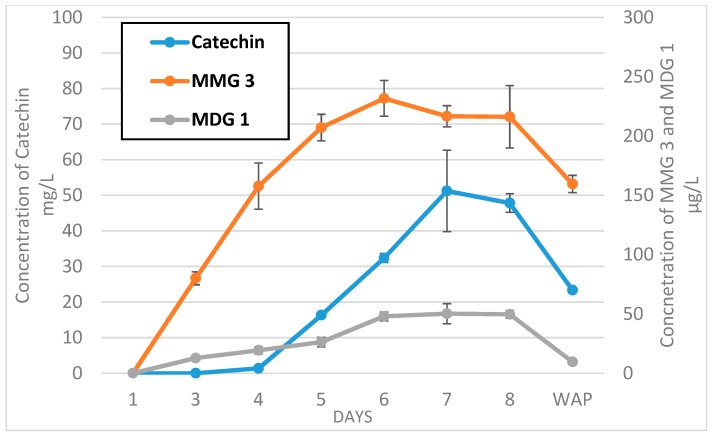
Evolution of catechin, MMG3, and MDG1 during GRE fermentation.

The extraction kinetics of mono- and diglycosylated flavanols are presented in Figure 2, Figure 3 and Figure 4. The compounds analyzed were isomers of monomers of (epi) catechin monoglycosides (MMG), dimers of (epi) catechin monoglycosides (DMG), monomers of (epi) catechin diglycosides (MDG), and dimers of (epi) catechin diglycosides (DDG).

### 2.1. Monomers of (epi) Catechin Monoglycosides (MMG) and Dimers of (epi) Catechin Monoglycosides (DMG)

#### 2.1.1. Extractions Kinetics of MMG and MDG during Grenache and Syrah Fermentation

The evolution kinetics of MMG concentrations during fermentation are represented in Figure 2A (Grenache) and Appendix B (Syrah). Those of DMG concentration fermentation are represented in Figure 2B (Grenache) and Appendix C (Syrah). It may be observed that the profiles of both MMG and DDG were similar for both Grenache (GRE) and Syrah (SYR) wines. The extraction of flavanol glycosides from grape solids in wine continued until day 5 for GRE and day 4 for SYR. The concentrations of monoglycosides were lower again in both wines at the end of AF, and further decreased in GRE wine after pressing. These observations confirm the findings in previous reports, that the concentration of phenolic acids, anthocyanins, and flavanols increased from the start of AF, reaching a maximum after approximately six days, followed by a reduction after pressing [10,20]. These observations were explained by further chemical interactions with other compounds, or adsorption onto grape tissues or yeasts, and has also been suggested for other varieties such as Monastrell [10]. In this study, the concentrations of monomers and dimers of (epi) catechin monoglycosides (MMG and DMG) in SYR sharply decreased after day 7 before pressing (Appendix B and Appendix C). These reductions might be explained by the presence of glycosidases in the cell walls of grape skin cells, which would produce (epi) catechin monomers and dimers.

#### 2.1.2. Grape Origins of MMG and DMG in Wine

**Figure 5:**
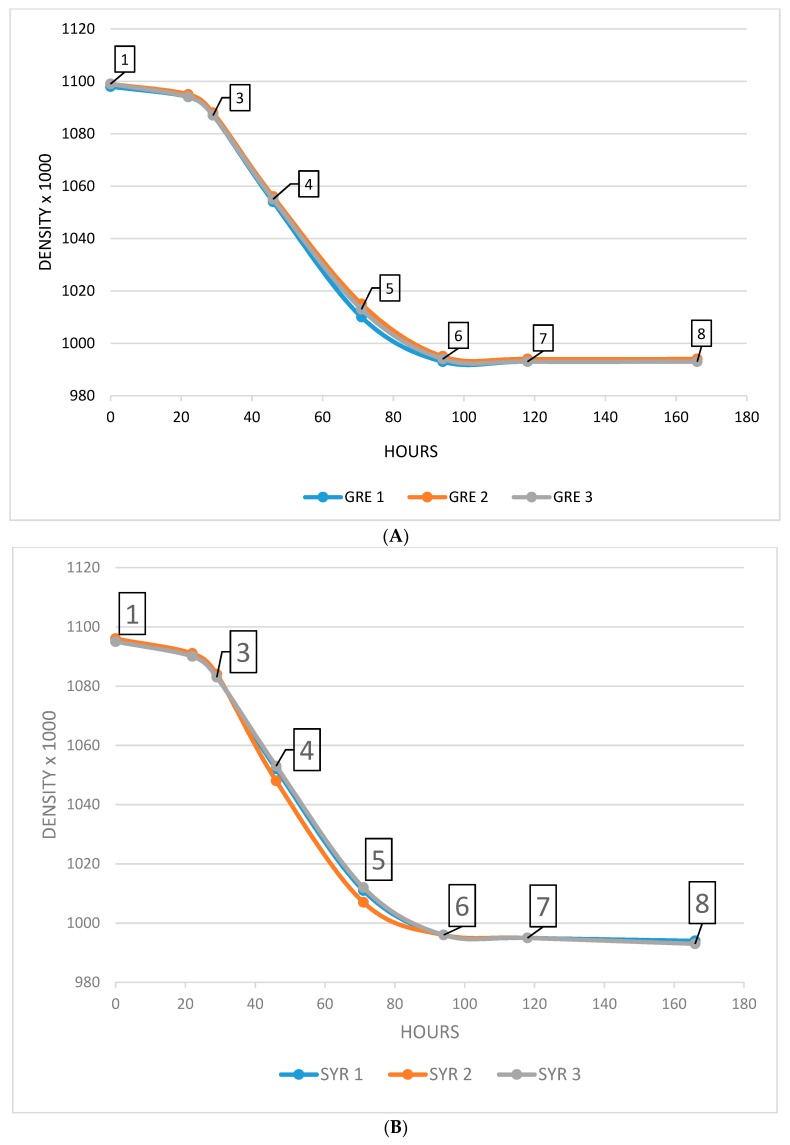
Samples during fermentation kinetics of GRE, GRE1, 2, 3 (**A**) and of Syrah (SYR) SYR1, 2, 3 (**B**).

The concentrations of three MMG isomers present in both varieties are shown in Table 1. Values are given for grape seeds, grape skin, and during winemaking at wine fermentation day 4 (WFD4), day 7 (WFD7), and after pressing (WAP). At WFD4, 75% of the initial sugar present was converted into ethanol (Material and Methods section, Figure 5). 

The results show that the distributions of each of the three isomers were in the same order of magnitude in skins, seeds, and at each of the three wine sampling dates for both varieties. It may be observed that isomer MMG3 was present at higher concentrations as compared to the other isomers in both skins, seeds, and during fermentation, where amounts were similar on WFD4 and WFD7, particularly in the case of Grenache. Table 1 also shows that isomer MMG1 was present at concentrations significantly lower than isomer MMG3 at all sampling points for both varieties. Notably, MMG2 was present at WFD4, but not in skins or seeds, indicating that this isomer may be formed during fermentation. A possible explanation for the fact that isomers 1 and 2 were no longer detectable at WFD7 and wine after pressing (WAP) is that they may be substrates for specific grape or yeast glycosidases.

Eleven DMG were determined in grapes and during fermentation (Table 2). The concentrations of the isomers were extremely variable, ranging from below the limit of detection to a maximum of 0.345 µg/g of grape skin. During winemaking, their concentrations varied from less than 10 µg to over 1 mg/L of wine. Concentrations in WAP were between less than 10 and over 100 µg/L. GRE wines contained higher concentrations of DMG than SYR wines. Isomer concentrations of DMG1, 2, 5, 6, and 7 s tended to increase at WFD4 to WAP for both varieties, whereas those of DMG3, 4, 8, 9, 10, and 11 decreased at the end of AF. These losses could be attributed, in part, to adsorption onto the solid components. However, it is more likely they were degraded by glycosidases.

### 2.2. Monomers of (epi) Catechin Diglycosides (MDG) and Dimers of (epi) Catechin Diglycosides (DDG)

#### 2.2.1. Extractions Kinetics of MDG and DDG during GRE and SYR Fermentation

Changes in the concentrations of MDG and DDG during GRE fermentation are shown in Figure 3A,B, respectively. The results for SYR are presented in Appendix D and Appendix E, respectively.

The evolution profiles of MDG and DDG were very similar to those of MMG and DMG during the fermentation of both GRE and SYR; the amounts of MDG and DDG increased during fermentation up to WFD7 and then decreased sharply at the end of fermentation. Reductions in the concentrations of MMG and DMG were observed at WFD7 for SYR and from WFD7 to WAP for GRE. MDG values were approximately 0.07 mg/L in both wines. Also, the concentrations of DDG were lower in GRE and SYR wines at approximately 0.014 and 0.04 mg/L, respectively.

#### 2.2.2. Grape Origins of MDG and DDG in Wines

Table 3 and Table 4 show the concentrations of (epi) catechin monomeric (MDG) and dimeric diglycoside (DDG) isomers in grape seeds and skin and wines during fermentation at WFD4, WFD7, and in WAP. Measurable amounts of four MDG and DDG isomers were present in both SYR and GRE seeds. Isomer MDG1 was found at all stages of fermentation, with a reduction in its concentration after WAP. Likewise, MDG2 and 3 were found in GRE up to WFD7.

DDG occurred in seeds but not in skins for both SYR and GRE varieties; the levels of isomer concentration in DDG3 and 4 increased between WFD4 and WFD7, and then decreased to negligible values for GRE. These compounds were not detected for SYR at WFD7 on in WAP.

### 2.3. Evolution between Monomer and Dimer Concentrations during Fermentation

As Figure 2 and Figure 3 and Appendix B, Appendix C, Appendix D and Appendix E illustrate, similar evolutions were observed for the extraction kinetics of the studied compounds. It is important to point out that for all monoglycosides and diglycosides, in addition to each of their 22 isomers, changes in the direction of concentration trends occurred precisely on the seventh day of fermentation for SYR and on the eighth day for Grenache.

Similar trends were observed for all the diglycosides and isomers MMG3, and DMG3/4, 9, 10/11, in that concentrations increased up to the seventh day of fermentation for SYR to the eighth day for Grenache. In both cases, levels had diminished by 50% or more by the end of fermentation. Concentrations in SYR wine after pressing remained stable. It is clearly illustrated in Figure 5 that the concentration trend for catechin is very closely mirrored by that of isomer MMG3, and to a lesser extent, MDG1.

Concentrations of the other DMG (1, 2, 5/6, 7, 8) isomers increased up to day 4 of fermentation, and remained stable up to day 7 (SYR) and day 8 (GRE) before further increasing at the end of fermentation and in wine after pressing (Appendix C).

## 3. Materials and Methods

### 3.1. Reagents, Standards and Calibration

Deionized water was purified with a Milli-Q purification system (Millipore, Molsheim, France). Formic acid and HPLC-grade methanol were purchased from Sigma Aldrich (St Louis, MO, USA). (+)-catechin was purchased from Sigma Chemical Company (St Louis, MO, USA). The (+)-catechin 4′-*O*-β-glucoside (C4′OG) was hemi-synthesized as described previously [21]. 

Calibration curves in the range of 0.025 to 5 mg·L^−1^ were prepared in a mixture of methanol/water (5:5) for C4′OG. Flavanol mono- and diglycosides (monomers, dimers) were semi-quantified C4′OG by multiple reaction monitoring (MRM).

### 3.2. Grape and Wine Samples

#### 3.2.1. Grape Samples

Samples of GRE and SYR grapes were harvested from Unité Expérimentale de Pech Rouge in Gruissan (France) on 13 and 28 August 2017, respectively, and were stored at −80 °C. The grapes were defrosted for 16 h before the start of the fermentation on 16 April 2018. 

The seeds and skins of 100 berries were manually removed on ice. Ten grams of each tissue were extracted with 200 mL of acetone/water (7:3) overnight under nitrogen with mechanical stirring. Solutions were filtered on filter paper (N°3 prat-dumas, Couze-St-Front, France), evaporated under reduced pressure at 37 °C with a rotary evaporator (Buchi R3, Rungis, France). The residues were dissolved in deionized water and freeze dried at −60 °C (Crios, Crytotec., St Gely du Fesc, France). The resulting tannin extract powders were stored at −20 °C. All experiments were performed in triplicate.

#### 3.2.2. Winemaking and Samples

All winemaking procedures were carried out in triplicate for both grape varieties. After manual destemming and crushing, musts (characteristics in Appendix A) were inoculated with yeasts (OKAY, 20 g·hL^−1^) and supplemented with DAP (di-ammonium phosphate, 150 mg·L^−1^). Fermentations were performed using 1 L “French Press” coffee plungers (small-lot fermentation adapted from the Australian Wine Research Institute (AWRI) method [22] at 22 °C over eight days. Cap management was carried out every day during AF by submerging pomace with the plunger. The density was monitored with a portable densimeter (DMA 35, Anton Paar, Les Ulis, France). At the end of AF, wine was pressed and the classical wine chemical parameters were determined (data not shown). 

Seven samples were taken during fermentation (Figure 5). Five milliliters of each sample were centrifuged (5 min, 5000 rpm), frozen, and stored at −85 °C before analysis. 

### 3.3. UHPLC-MS/MS

A UHPLC Nexera X2 UHPLC system was used with two reversed-phase columns in series for separation: Zorbax SB AQ (2.1 × 150 mm and 2.1 × 100 mm 1.8 μm from Agilent Technologies, Santa Clara, CA, USA)**.** MS/MS experiments were carried out using the Shimadzu UHPLC system described above coupled with a Shimadzu LCMS-8050 triple quadrupole mass spectrometer using the multiple reaction monitoring (MRM) technique operating in negative ion mode. All UHPLC and MS/MS parameters were identical, as previously described [21]. The average and standard deviations of the chemical analyses were performed on Excel 2013.

## 4. Conclusions

This study reports the kinetics of extraction of monomeric and dimeric flavanols of mono- and diglycosides in GRE and SYR grape berries, and for the first time, in wine during fermentation. Data were obtained for a total of 22 isomers using a new UHPL-MS technique described in previous work [21]. 

For all monoglycosides and diglycosides, in addition to each of their 22 isomers, changes in the direction of concentration trends occurred precisely on the seventh day of fermentation for SYR and on the eighth day for GRE. Certain compounds were specific to either grape skins (as DMG isomers) or to seeds (as DDG isomers). The extraction kinetics profiles of total glycosides were similar for both grape varieties. An increase in concentration during fermentation was observed, followed by a degradation of specific compounds at the end of fermentation; the latter observations may be explained by the activity of glycosidases extracted during fermentation and pressing. MMG3 was the major isomer of glycosides in grape skin and seeds and was present during fermentation as well as in GRE and SYR WAP.

Further studies using isolation techniques or hemisynthesis are currently in progress to identify the structure of these new glycosides. Their potential roles as PA precursors and extractions skin markers during fermentation will also be investigated.

## Figures and Tables

**Table 1:** Monomers of (epi) catechin monoglycoside (MMG) isomers measured in grape seed and skin, and during winemaking at wine fermentation day 4 (WFD4), day 7 (WFD7), and wine after pressing (WAP).

MMG	Grenache					Syrah				
	Seed ^a^	Skin ^a^	WFD4 ^b^	WFD7 ^b^	WAP ^b^	Seed ^a^	Skin ^a^	WFD4 ^b^	WFD7 ^b^	WAP ^b^
**1**	0.002 ± 4.10 × 10^−4^	n.d.	2.27 ± 0.475	n.d.	n.d.	0.002 ± 7.10 × 10^−4^	0.007 ± 0.02	4.44 ± 0.739	n.d.	n.d.
**2**	n.d.	n.d.	16.6 ± 2.30	n.d.	n.d.	n.d.	n.d.	87.7 ± 2.46	n.d.	n.d.
**3**	0.205 ± 0.039	0.643 ± 0.102	207 ± 11.1	216 ± 26.3	160 ± 7.27	0.045 ± 0.005	0.307 ± 0.056	80.7 ± 4.40	62.7 ± 2.17	56.8 ± 1.57

^a^ µg/g of grape tissue, ^b^ µg/L, n.d. not detected. Results are the average of three biological replicates with the corresponding standard deviations.

**Table 2:** Contents of dimeric (epi) catechin monoglycoside (DMG) isomers measured in grape seed and skin, and during winemaking at WFD4, WFD7, and WAP.

DMG	Grenache					Syrah				
	Seed ^a^	Skin ^a^	WFD4 ^b^	WFD7 ^b^	WAP ^b^	Seed ^a^	Skin ^a^	WFD4 ^b^	WFD7 ^b^	WAP ^b^
**1**	n.d.	0.015 ± 0.006	2.99 ± 1.04	5.24 ± 2.06	3.17 ± 0.831	n.d.	0.028 ± 0.004	6.41 ± 0.136	3.79 ± 0.480	3.90 ± 0.378
**2**	n.d.	7.10 × 10^−4^ ± 2.10 × 10^−4^	5.00 ± 1.53	13.4 ± 6.36	16.3 ± 0.529	n.d.	0.004 ± 0	3.03 ± 0.390	8.15 ± 1.83	7.18 ± 1.66
**3/4**	n.d.	0.008 ± 0.003	8.50 ± 0.943	28.0 ± 3.08	n.d.	n.d.	0.015 ± 0.004	6.80 ± 2.14	4.86 ± 0.934	6.85 ± 1.09
**5/6**	n.d.	0.009 ± 0.003	3.62 ± 0.852	15.0 ± 0.395	49.4 ± 4.05	n.d.	0.015 ± 0.002	4.78 ± 0.317	15.2 ± 1.82	16.0 ± 0.791
**7**	n.d.	0.022 ± 0.001	18.1 ± 1.60	24.9 ± 1.2	70.1 ± 2.87	n.d.	0.027 ± 0.009	10.9 ± 1.05	21.8 ± 0.962	24.5 ± 0.106
**8**	n.d.	0.024 ± 0.004	n.d.	3.12 ± 0.569	n.d.	n.d.	0.078 ± 0.012	8.06 ± 2.37	n.d.	n.d.
**9**	n.d.	0.041 ± 0.019	15.0 ± 1.19	23.4 ± 4.09	n.d.	n.d.	0.031 ± 0.004	6.67 ± 2.08	n.d.	n.d.
**10/11**	n.d.	0.345 ± 0.114	75.0 ± 6.02	114.6 ± 5.62	n.d.	n.d.	0.250 ± 0.033	34.4 ± 3.30	n.d.	n.d.

^a^ µg/g of grape tissue, ^b^ µg/L, n.d. not detected. Results are the average of three biological replicates with the corresponding standard deviations.

**Table 3:** Monomeric (epi) catechin diglycoside (MDG) isomers concentrations measured in grape seeds and skins, and during winemaking at WFD4, WFD7, and WAP.

MDG	Grenache					Syrah				
	Seed ^a^	Skin ^a^	WFD4 ^b^	WFD7 ^b^	WAP ^b^	Seed ^a^	Skin ^a^	WFD4 ^b^	WFD7 ^b^	WAP ^b^
**1**	0.172 ± 0.014	0.038 ± 0.014	26.31 ± 3.97	49.7 ± 3.28	9.55 ± 0.014	0.122 ± 0.009	0.048 ± 0.013	39.5 ± 4.83	2.66 ± 0.959	3.09 ± 0.016
**2**	0.243 ± 0.014	n.d.	3.84 ± 0.947	26.6± 0.197	n.d.	0.136 ± 0.007	n.d.	9.48 ± 0.680	n.d.	n.d.
**3**	0.342 ± 0.026	n.d.	n.d.	8.10 ± 0.696	n.d.	0.178 ± 0.018	n.d.	n.d.	n.d.	n.d.
**4**	0.250 ± 0.058	n.d.	n.d.	n.d.	n.d.	0.074 ± 0.010	n.d.	7.54 ± 2.77	n.d.	n.d.

^a^ µg/g of grape tissue, ^b^ µg/L, n.d. not detected. Results are the average of three biological replicates with the corresponding standard deviations.

**Table 4:** Dimeric (epi) catechin diglycoside (DDG) isomers measured in grape seeds and skin, and wine during fermentation at WFD4, WFD7, and WAP.

DDG	Grenache					Syrah				
	Seed ^a^	Skin ^a^	WFD4 ^b^	WFD7 ^b^	WAP ^b^	Seed ^a^	Skin ^a^	WFD4 ^b^	WFD7 ^b^	WAP ^b^
**1**	0.032 ± 0.002	n.d.	n.d.	n.d.	0.610 ± 0.061	0.015 ± 0.005	n.d.	n.d.	n.d.	n.d.
**2**	0.079 ± 0.006	n.d.	0.716 ± 0.15	5.74 ± 0.951	0.110 ± 0.018	0.041 ± 0.005	n.d.	0.926 ± 0.107	n.d.	n.d.
**3**	0.043 ± 0.003	n.d.	0.672 ± 0	2.790 ± 0.297	1.070 ± 0.035	0.029 ± 0.005	n.d.	0.123 ± 0	n.d.	n.d.
**4**	0.080 ± 0.018	n.d.	2.23 ± 0.004	6.350 ± 0.953	n.d.	0.042 ± 0.005	n.d.	1.300 ± 0.336	n.d.	n.d.

^a^ µg/g of grape tissue, ^b^ µg/L, n.d. not detected. Results are the average of three biological replicates with the corresponding standard deviations.

**Figure A1:**
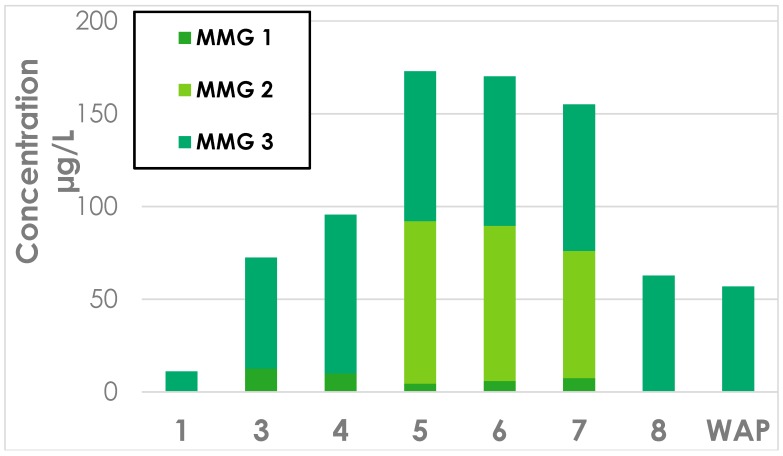
Evolution of MMG isomers during Syrah fermentation.

**Figure A2:**
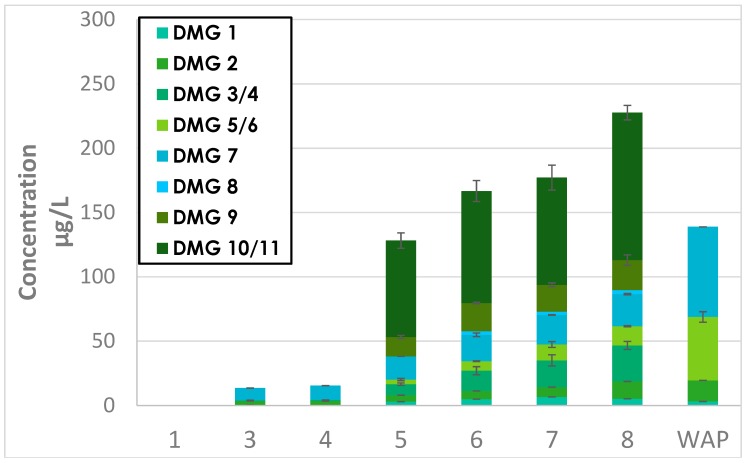
Evolution of DMG isomers during Syrah fermentation.

**Figure A3:**
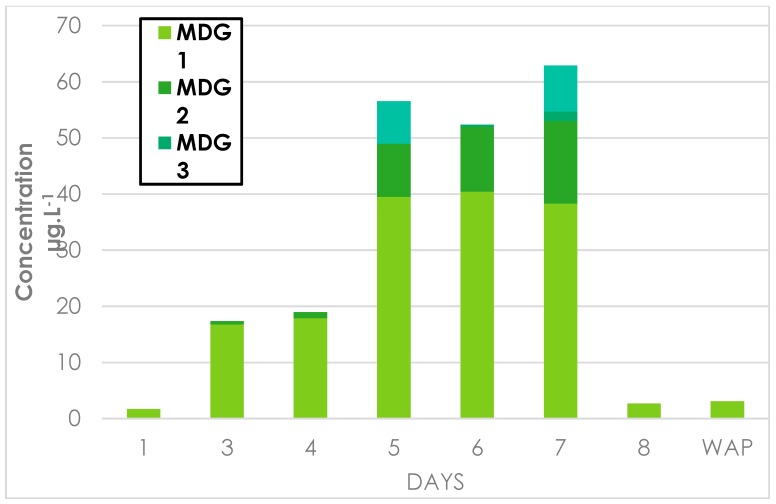
Evolution of MDG isomers during Syrah fermentation.

**Figure A4:**
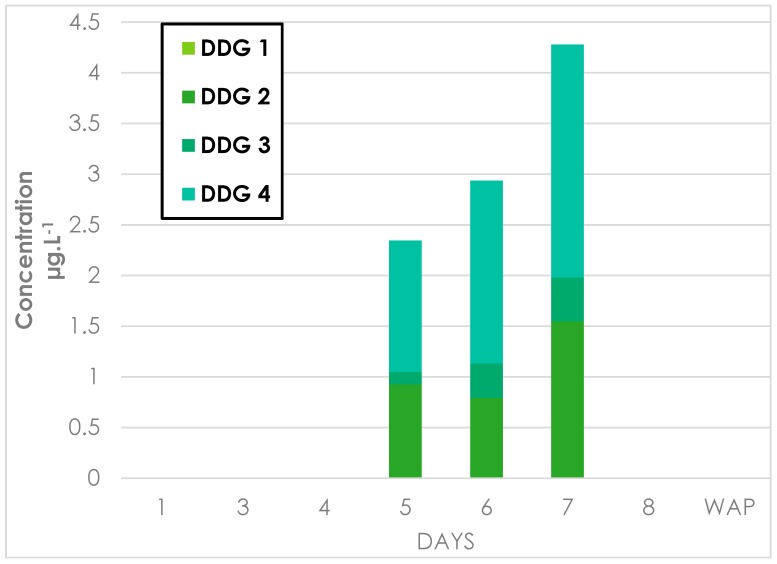
Evolution of DDG isomers during Syrah fermentation.

## Appendix A

**Table A1:** Must characteristics.

Grape Variety	Berry Weight (g)	Brix	Sugars g·L^−1^	Potential Alcohol by Volume %vol
**GRE 1**	584	23.2	228.7	13.59
**GRE 2**	586	23.2	228.7	13.59
**GRE 3**	578	23.2	228.7	13.59
**SYR 1**	561	22.0	214.8	12.76
**SYR 2**	550	22.0	214.8	12.76
**SYR 3**	536	22.0	214.8	12.76
